# Analysis of DNA methylation patterns associated with the gastric cancer genome

**DOI:** 10.3892/ol.2014.1838

**Published:** 2014-01-29

**Authors:** YI CHENG, ZHI YAN, YIN LIU, CHENGBAI LIANG, HONG XIA, JUNMING FENG, GUORONG ZHENG, HESHENG LUO

**Affiliations:** 1 Department of Gastroenterology, Remin Hospital of Wuhan University, Wuhan, Hubei 430060, P.R. China; 2 Department of Gastroenterology, Wuhan General Hospital of Guangzhou Command, Wuhan, Hubei 430070, P.R. China; 3 Department of Pathology, Wuhan General Hospital of Guangzhou Command, Wuhan, Hubei 430070, P.R. China

**Keywords:** gastric cancer, DNA methylation, genome-wide, methylation microarray

## Abstract

The objective of the current study was to investigate the characteristics of DNA methylation patterns associated with the gastric cancer genome and to identify clinically useful diagnostic markers and therapeutic targets for gastric cancer. The Infinium 450K methylation microarray was used to compare differential DNA methylation sites of gastric cancer tissue with that of normal gastric tissue. The results of the DNA microarray analysis were confirmed by pyrosequencing. Functional analysis of the differential genes was performed using the GO software. The effect of candidate site methylation on gene expression was monitored using quantitative polymerase chain reaction analysis. Of the 2,645 differential methylation sites identified in gastric cancer tissues, 2,016 were hypermethylated sites, 629 were hypomethylated sites, 826 were located in promoter regions and 1,024 were located within genes. These differential sites were associated with 1,352 genes. In total, five sites were selected and pyrosequencing verified the results of the microarray analysis in five of the sites. Change in gastric cancer DNA methylation pattern was a common occurrence. Differential methylation sites appeared more often in non-promoter regions. The associated genes were involved in multiple signaling pathways, and hypermethylated and hypomethylated sites were involved in roughly the same signaling pathways. Methylation of the genome promoted gene expression. *TRIM15, ITGAM, MSX2* and *FAM38A* may be candidate genes for diagnosing gastric cancer.

## Introduction

Gastric cancer is a malignant disease with high incidence and mortality rates, particularly in Asian populations (1,2). The oncogenesis and development of gastric cancer are influenced by genetic and epigenetic factors. DNA methylation is the most studied epigenetic mechanism. At present, the focus of the majority of studies is the inactivation of gene expression by hypermethylation of DNA located in tumor suppressor gene promoter regions. The observation that promoter CG island methylation inactivates a number of tumor suppressor genes in gastric cancer, such as *RASSF1A, P16 a*nd *E-cadherin*, has increased the understanding of the mechanism of gastric carcinogenesis (3). For technical reasons, the majority of previous studies have focused on only a few genes. With the development of sequencing and microarray technology, a large-scale study of the pattern of DNA methylation, which is also called the methylome, became possible (4,5). The present study analyzed DNA methylation characteristics of the gastric cancer genome using the Infinium 450K methylation microarray to further understand the pathogenesis of gastric cancer and identify potential therapeutic targets and diagnostic markers for gastric cancer.

## Materials and methods

### Clinical samples

In total, 40 patients with gastric cancer from the Wuhan General Hospital of the Guangzhou Command (Wuhan, China) during the period between March 2011 and June 2012 were included. Each tumor sample was matched with adjacent apparently normal mucosa (3–5 cm from the tumor margin) removed during the same surgery. All samples were collected by one surgical pathology fellow from the operating room immediately following the surgical resection and frozen in the liquid nitrogen. Pathological diagnosis was determined independently by two pathologists and disagreement was resolved by consensus. The study protocol was approved by the ethics committee of Wuhan General Hospital of Guangzhou Command (Wuhan, China).

### Genomic DNA extraction and quality control

DNA was extracted from ~25 mg of tissue using a DNA extraction kit (Quick-gDNA MiniPrep; Zymo Research Corporation, Orange, CA, USA) according to the manufacturer’s instructions, and DNA quality was assessed using spectrophotometry (UV-100, Shanghai Precision Instrument Co., Ltd., Shanghai, China) and agarose gel electrophoresis (Amresco LLC, Solon, OH, USA).

### DNA methylation profiling with Infinium 450K methylation assay

In total, six paired samples were processed on the chip (12 samples/chip). Genomic DNA (500 ng) was treated with sodium bisulfite using the Zymo EZ DNA Methylation Kit™ (Zymo Research Corporation) according to the manufacturer’s instructions, with the alternative incubation conditions recommended for the Illumina Infinium 450K methylation assay (Illumina Inc., San Diego, CA, USA). The methylation assay was performed on 4 μl of bisulfite-converted genomic DNA at 50 ng/μl according to the Infinium HD methylation assay (Illumina Inc.) instructions. The quality of the results was assessed using the GenomeStudio™ Methylation Module v1.8 software (Illumina, Inc., San Diego, CA, USA) and all samples passed this quality control. β-values were extracted using the same software.

### Bisulfite pyrosequencing

A total of six CpGs sites were selected for technical validation of Infinium 450K methylation by the bisulfate pyrosequencing technique on 40 paired samples (cancer and normal tissues). One candidate site in each of the five genes (*TRIM15, ITGAM, SLMO2, MSX2* and *FAM38A*) was selected and all were located in the gene body. Primers for polymerase chain reaction (PCR) amplification and sequencing were deduced using the PyroMark Assay Design 2.0 software (Qiagen, Hilden, Germany) and all primer sequences are shown in Table I. Bisulfite conversion of genomic DNA was performed, followed by PCR. PCRs were performed under the following conditions: 95°C for 15 min; 45 cycles of 94°C for 30 sec, 56°C for 30 sec, 72°C for 30 sec; and 72°C for 10 min. The success of amplification was assessed by agarose gel electrophoresis and pyrosequencing of the PCR products was performed using the PyromarkID system (Qiagen). Only blue values (perfect calls) were considered for subsequent analyses.

### RNA extraction and quantitative PCR (qPCR)

Total cellular RNA was extracted using the TRIzol method. RNA quality was assessed by spectrophotometry and agarose gel electrophoresis. A total of 5 μg of RNA was reverse transcribed using the All-in-One™ First Strand cDNA synthesis kit (AORT-100; GeneCopoeia™, Rockville, MD, USA). Quantitative qPCR amplification reactions were performed using the All-in-One qPCR master mix (AOPR-1200; GeneCopoeia) with a Real-Time PCR system (ABI StepOne plus; Applied Biosystems, Carlsbad, CA, USA). The expression levels of the genes were normalized to the expression of actin. Primer sequences and PCR conditions are shown in Table II.

### Bioinformatics analysis

Integrated gene ontology and pathway analysis database MAS3.0 (http://www.capitalbio.com) were to investigate potential molecular function and the pathway of the candidate biomarkers.

### Statistical analysis

All statistical analyses were performed using SPSS 13.0 for Windows (SPSS, Inc., Chicago, IL, USA). Group comparisons were performed using a paired samples t-test. Two-sided P<0.05 was considered to indicate a statistically significant difference.

## Results

### Patients

The present study was conducted on 40 patients (23 male and 17 female) with gastric cancer, with a mean age of 56 years. The methylation microarray procedure was performed on specimens from six patients. The tumors were all adenocarcinomas.

### Pyrosequencing

**Figure 1:**
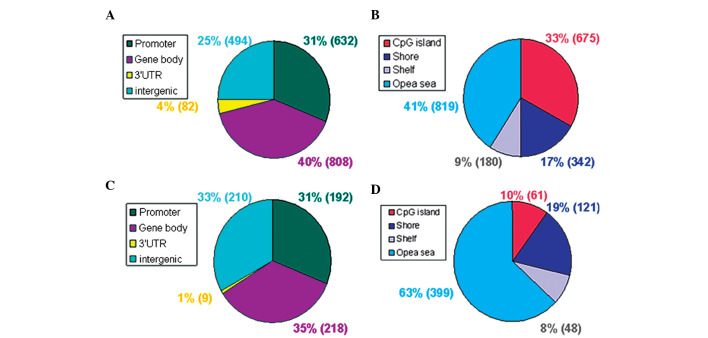
Distribution of the differential methylation sites. (A) Hypermethylated sites were classified into the following functional genomic groups: Promoter, gene body, 3′ untranslated region and intergenic. (B) Hypermethylated sites were classified in terms of CpG content, as follows: Island, shore, shelf and ‘open sea’. (C) Hypomethylated sites classified into functional genomic groups and (D) hypomethylated sites classified by CpG content.

**Figure 2:**
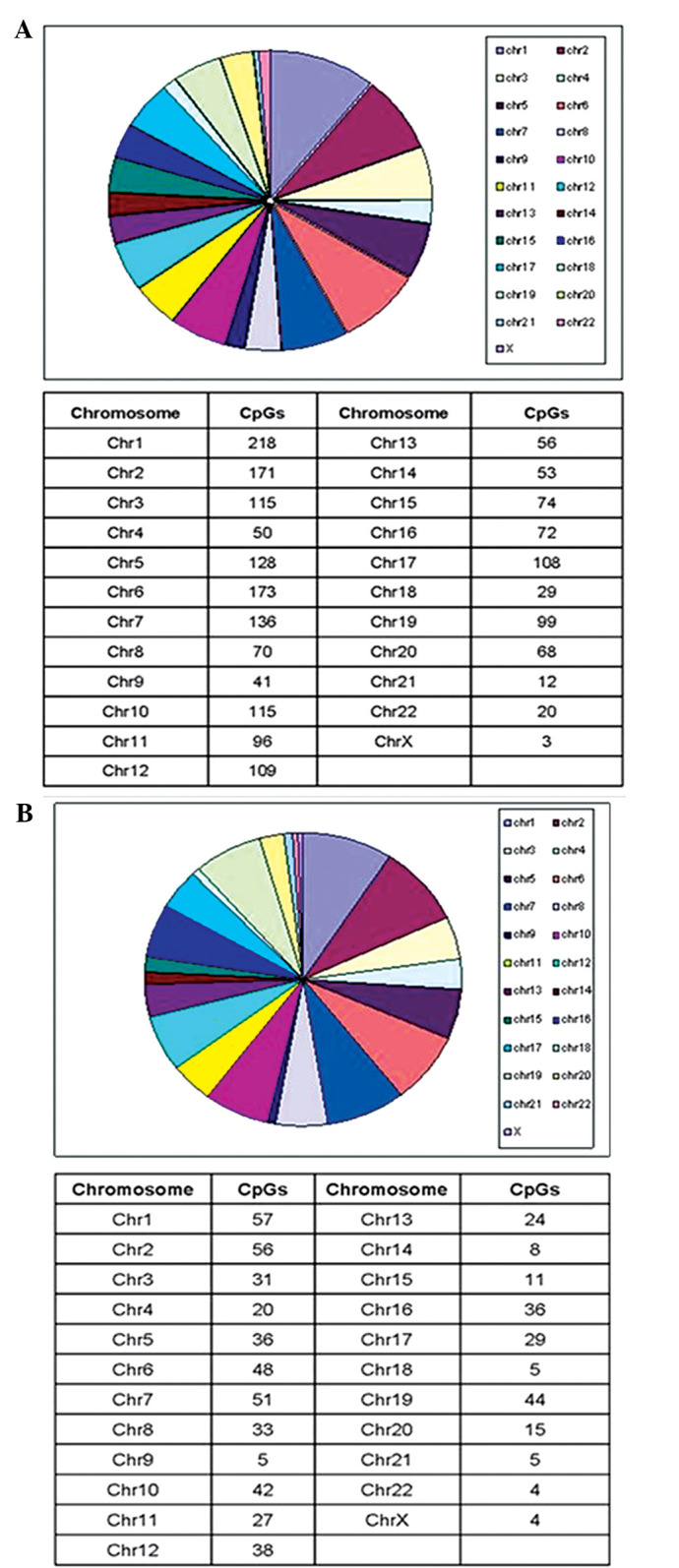
Chromosome locations of the differential methylation sites. (A) Hypermethylated and (B) hypomethylated sites.

Pyrosequencing was performed on specimens from 40 patients. In total, 2,645 methylation differential sites (covering 1,352 genes) were detected in the gastric cancer tissues compared with the normal tissues. In the gastric cancer tissue, 2,016 sites (covering 1,008 genes) were hypermethylated and 629 sites (covering 344 genes) were hypomethylated (Table III). From the functional genome distribution standpoint, 824 sites (31%) were located in promoters and 1,026 sites (39%) are located in the gene body. In addition, 91 sites (3%) and 704 sites (27%) corresponded to the 3′-untranslated regions and intergenic sequences, respectively. From the CpG content and neighborhood context, 736 CpG sites (28%) were in CpG islands, 463 (17%) were in CpG shores, 228 (9%) were in CpG shelves and 1,218 (46%) were in the ‘open sea’ (isolated CpGs in the genome). The methylation differential sites were distributed among all 22 autosomal chromosomes and one sex chromosome. The majority of positions were harbored in chromosome one (9.1%), followed by chromosomes two (8.9%) and six (7.7%). The distribution of these sites is shown in Figs. 1 and 2.

### Gene regulation

A total of 979 genes were upregulated and 314 genes were downregulated in gastric cancer samples compared with the normal samples. Signaling pathway analyses showed that the majority of the genes that were upregulated and downregulated in gastric cancer were involved in the same pathways, including apoptosis, cell cycle, ErbB, Janus kinase-signal transducer and activator of transcription, mitogen-activated protein kinases, p53, transforming growth factor-β, Toll-like receptor, vascular endothelial growth factor and Wnt signaling pathways. We proposed that these pathway alterations may be associated with the clinical pathological features and outcome of GC patients. An integrated gene ontology database was used to annotate the molecular function of the differentially expressed genes. The results showed that genes that were upregulated and downregulated in gastric cancer were involved in the majority of the important biological process associated with human cancer, including regulation of the inhibitor-κB kinase/nuclear factor-κB cascade, cell differentiation, cell cycle arrest, caspase activation and cell proliferation (Table IV).

### Methylation levels

**Figure 3:**
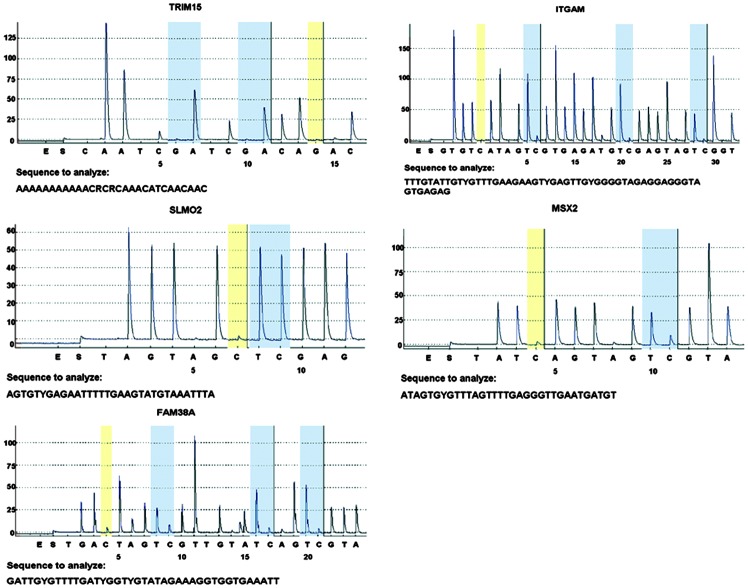
Pyrosequencing for five sites.

In total, five differential sites (*TRIM15, ITGAM, SLMO2, MSX2* and *FAM38A*) were selected and verified in 40 samples by pyrosequencing. The mean methylation levels of gastric cancer tissues were higher than those of normal gastric tissues for two sites (*TRIM15* and *ITGAM*) and lower for two sites (*MSX2* and *FAM38A*), which were consistent with the results of the microarray analysis. The mean methylation level of *SLMO2* was not different between normal and cancer tissues (Table V, Fig. 3).

### Transcriptional levels

To examine the transcriptional level of the four genes (T*RIM15, IGTAM, MSX2* and *FAM38A*), qPCR was performed using primers specific for these genes. qPCR results showed that the expression levels of ITGAM, MSX2 and FAM38A were upregulated in gastric cancer tissues, while the expression level of TRIM15 was downregulated (P<0.05).

## Discussion

DNA methylation is important in the development of gastric cancer. Previously, it has been found that hypermethylation inactivates a number of gene promoters. However, previous studies have been limited by technology that only allows analysis of a few genes. In addition, the majority of studies have paid close attention to the CpG island in the promoter region. According to current knowledge, only a few CpG dinucleotides have been identified at the promoter CpG islands, mostly scattered in the genome. In tumorigenesis, the importance of scattered CpG dinucleotide methylation remains poorly understood (6,7). Therefore, understanding genome-wide DNA methylation changes is necessary for the in-depth investigation of tumor occurrence.

The latest generation of methylation microarrays includes the Infinium 450K methylation microarray. This microarray detects >450,000 methylation sites per sample. It includes methylation regions, such as CpG islands, CpG shores, CpG sites outside of CpG islands, non-CpG methylated sites identified in human stem cells, differentially methylated sites identified in tumor versus normal tissues (multiple forms of cancer), across several tissue types, CpG islands outside of coding regions, miRNA promoter regions and disease-associated regions identified through genome-wide association study (8). To date, the Infinium 450K methylation microarray is the most attractive, powerful and cost-effective tool available for generating quantitative DNA methylomes in healthy and diseased individuals (9,10). Using the Infinium 450K methylation microarray, the present study compared the genomic DNA methylation of gastric cancer with that of normal gastric tissue, screened 2,645 differential sites, showed the detailed distribution of these differential sites and established a gastric cancer DNA methylation profile. Verification of the microarray results by pyrosequencing showed that these results were reliable.

A considerable number of differentially methylated sites are located in the promoter region. However, the majority of them appear within the gene body. A number of previous studies have shown abnormal methylation of the CpG island in the promoter region and increasing attention has been paid to methylation of the gene body (11,12). Unlike the correlation between promoter DNA methylation and gene transcription inhibition, the correlation between gene body methylation and gene expression is more complicated. A meta-analysis of this correlation by Jjingo *et al* (13) suggested that the gene body DNA methylation is highest when gene expression is moderate. Additionally, when gene expression is high or low, the degree of gene body DNA methylation is extremely low. In the current study of the correlation between methylation changes in four selected differentially methylated sites in the genome and gene expression, the effects of DNA methylation on gene expression varied between genes.

The current study found that the methylation changes in certain genes occurred in multiple sites, some in the promoter and some in the gene itself; certain sites became hypermethylated and others hypomethylated, which suggested that gene expression is regulated by complicated patterns of multi-site methylation. Bioinformatics analysis suggested no difference between genes with hypermethylated sites and genes with hypomethylated sites in associated signaling pathways, which included signaling pathways involved in apoptosis, cell proliferation and cell cycle control.

The present relatively large-scale investigation of methylation changes of gastric cancer, covering a relatively large genomic area, found a number of new differentially methylated sites, including hypermethylation and hypomethylation sites. Analysis of the results identified *TRIM15, ITGAM, MSX2* and *FAM38A* as possible candidate sites clinically useful for the diagnosis and treatment of gastric cancer. In addition, a number of differentially methylated sites were identified in the microRNA gene. Further studies must be performed to explain this phenomenon.

## Figures and Tables

**Table I:** Primers for bisulfite pyrosequencing.

Gene	Sequence	Nt	Tm, °C	GC, %
TRIM15	F: GGTTTAATGGTAGGTTGTTTAAGT	24	58.4	33.3
	R: ATATACCTCACTAACTTCCTATCTT	25	58.2	32.0
	S: ATCCAAAATAATAACCCCT	9	45.2	31.6
ITGAM	F: GTTAAGTGTGGTTTGGGTAGAGTTT	25	59.1	40.0
	R: ACTACTATCCCTCTCACTACCCTCCTCTA	29	60.3	48.3
	S: GGGGATTTTTTTTATTTATTATGTT	25	45.0	20.0
SLMO2	F: GGGGATGAGTTAGGAAGAAGAGT	23	62.6	47.8
	R: AATCCCATTCATCACTAATCCATTTCAACT	30	58.9	33.3
	S: AGATAGTTTTAGGGAGATTG	20	46.3	35.0
MSX2	F: GTTTTTAATAGGGTGGAGAGAGATTG	26	60.3	38.5
	R: TACCCCCTAATTTCCCACC	19	58.4	52.6
	S: ATGGTTTTGTTTTGTTAATAAAAT	24	44.2	16.7
FAM38A	F: TGGGGTTTTTTGATTGTAAAAGT	23	58.3	30.4
	R: CTAAAAAATCTTCCCCAAATTTCACC	26	60.6	34.6
	S: GTTTGTTTGAGGTTTTTAGATA	22	44.1	27.3

F, forward; R, reverse; S, sequence to analyze; Nt, nucleotides; Tm, temperature; GC, gastric cancer.

**Table II:** Primers for quantitative polymerase chain reaction.

Gene	Forward primer	Reverse primer	Product, bp	Tm, °C
Actin	GTCCACCGCAAATGCTTCTA	TGCTGTCACCTTCACCGTTC	190	59
TRIM15	AGCAAGAAGCATCAGGTGGA	GACAAGGTCAGGAGAAATGGC	294	59
ITGAM	CCTTGTGGTTCCTCAGTGGT	CTTGGAAGGTCATTGCGTTT	154	59
MSX2	AAGATGGAGCGGCGTGGAT	CGAGGAGCTGGGATGTGGT	138	59
FAM38A	ATCCACTCCGGGGACTACTT	GGTAGCTGTCCTGCCTGTTC	197	59

bp, base pairs; Tm, temperature.

**Table III:** Hypermethylated and hypomethylated sites (partly).

A, Hypermethylated			

Target ID	UCSC name	UCSC refGene group	Chromosome
cg08977390	TRIM15	Body	6
cg21678445	ZNF521	Body	18
cg02256631	ITGAM	Body	16
cg01192077	EBF1	Body	5
cg18369516	ZBTB46	Body	20
cg06445348	ILDR2	Body	1
cg18125573	RARA	Body	17
cg04407470	NR2E1	Body	6
cg22388634	VSX1	Body	20
cg14063008	DAB2IP	Body	9
cg24171907	CNRIP1	5′UTR	2
cg16306190	LRRC34	1st exon	3
cg11595545	KCNA3	1st exon	1
cg08048222	ZNF671	TSS200	19
cg09734791	MSC	1st exon	8
cg25024074	ITGA4	1st exon	2
cg16964348	NPY	TSS200	7
cg17508991	HCK	TSS1500	20
cg18372896	JDP2	5′UTR	14
cg17219660	GPR37L1	TSS200	1

B, Hypomethylated			

Target ID	UCSC name	UCSC refGene group	Chromosome

cg20726575	SLMO2	Body	20
cg06013117	MSX2	Body	5
cg06007201	FAM38A	Body	16
cg21499869	ELL	Body	19
cg16499677	C14ORF37	Body	14
cg23263641	CADM4	Body	19
cg18847089	PRKAR1B	Body	7
cg27341866	C19orf35	Body	19
cg13826564	LTBP3	Body	11
cg04529865	GALNT9	Body	12
cg01515802	LATR1	TSS200	19
cg22888958	CREB5	5′UTR	7
cg23264429	STAMBPL1	5′UTR	10
cg19060371	LCP1	5′UTR	13
cg01318557	LAT2	5′UTR	7
cg22274117	ATXN1	5′UTR	6
cg06442489	ZSCAN18	TSS1500	19
cg02829601	SYTL3	TSS200	6
cg06523556	CHRNA6	TSS200	8
cg20117742	LAIR1	TSS200	19

UTR, untranslated region.

**Table IV:** Signaling pathway analyses.

Pathway	Upregulated	P-value	Downregulated	P-value
Apoptosis	BCL2, CAPN2, ENDOD1, NFKBIA, NTRK1 and PRKACB	9.77E-04	CASP8, IL1A, IL1B, PRKACA and TNFRSF10A	4.44E-05
Cell cycle	ANAPC5, CDH1 and MYT1	0.193209	CDC2, CDK1 and LAT	0.094077
ErbB	CAMK2B, GAB1, GRB2 and NRG2	0.02511	PRKACA and STAT5A	0.054748
Jak-STAT	CNTFR, CSF3R, GHR, GRB2, IFNK, IL22RA1, IL2RA, IL7 and PIAS4	1.77E-04	IL12B, IL21R, SOCS2, SOCS5 and STAT5A	5.93E-04
MAPK	CACNA2D2, CACNA2D3, DUSP16, FGF12, GNG12, GRB2, MAPKAPK2, MRAS, NTRK1, PDGFA, PRKACB and RASGRF1	2.37E-04	CACNA1C, CACNA1H, CACNA1I, DUSP14, HSPA1A, HSPA1A, IL1A, IL1B, IL1R2, MEF2C, NF1, PRKACA, PRKACA, RPS6KA2 and SCT	1.52E-12
p53	SESN1, SESN3 and TSC2	0.057786	CASP8, CDC2, CDK1 and RRM2	0.00338
TGF-β	BMP2, CHRD, GDF6, SMAD6, SMAD7, SMAD9 and SMURF2	1.25E-04	BMPR1B and INHBA	0.054748
Toll-like receptor	NFKBIA, TOLLIP and TRAF3	0.140739	CASP8, IL12B, IL1B and TLR6	0.001034
VEGF	MAPKAPK2 and NFATC1	0.251502	NFATC1 and PRKACA	0.043002
Wnt	CAMK2B, CTBP2, LRP5, NFATC1, POR, PPP2R5C, PRICKLE1, PRKACE, SFRP1, SOX17, TCF4, WNT11 and WNT6	8.63E-08	APC, NFATC1, PRKACA and PRKACA	0.004401

Jak-STAT, Janus kinase-signal transducer and activator of transcription; MAPK, mitogen-activated protein kinases; TGF-β, transforming growth factor-β; VEGF, vascular endothelial growth factor.

**Table V:** Level of methylation of five sites.

	Level of methylation, %		
		
Gene	GC tissues	Normal tissues	P-value
TRIM15	10.71±3.08	5.86±1.65	<0.0001
ITGAM	44.23±17.68	26.34±9.41	<0.0001
SLMO2	74.72±17.01	71.73±10.77	0.561
MSX2	21.34±7.49	38.20±6.49	<0.0001
FAM38A	18.21±5.43	32.92±6.71	<0.0001

GC, gastric cancer.
